# Comparisons on factors affecting residents fulfilling self-determination in ethno-specific and mainstream nursing homes: a qualitative study

**DOI:** 10.1186/s12877-023-03800-w

**Published:** 2023-02-07

**Authors:** Lily Xiao, Carolyn Gregoric, Sue Gordon, Shahid Ullah, Ian Goodwin-Smith, Eimear Muir-Cochrane, Sara Blunt

**Affiliations:** 1 College of Nursing and Health Sciences, Flinders University, Bedford Park, Adelaide, South Australia 5042 Australia; 2 Flinders University, Adelaide, Australia; 3 College of Medicine and Public Health, Flinders University, Adelaide, Australia; 4 College of Business, Government & Law, Flinders University, Adelaide, Australia; 5 Kalyra Communities, Adelaide, South Australia Australia

**Keywords:** Aged care, Culturally competent care, Nursing home, Person-centred care, Self-determination, Workforce issues

## Abstract

**Background:**

Studies revealed that supporting residents fulfilling self-determination is positively associated with their health, wellbeing and quality of life. Cross-cultural care poses significant challenges for nursing home residents to fulfil their self-determination in control of own care and maintaining meaningful connections with others. The aim of the study was to compare factors affecting residents fulfilling self-determination in ethno-specific and mainstream nursing homes.

**Methods:**

A qualitative descriptive approach was applied to the study. Culturally competent care and person-centred care were employed as guiding frameworks. Individual interviews or a focus group with residents and family members were conducted to collect data.

**Results:**

In total, 29 participants participated in the study. Three main themes were identified: communicating needs and preferences; mastering own care; and maintaining meaningful relationships. Each theme includes sub-themes that detail similarities and differences of factors affecting residents fulfilling self-determination in the two type nursing homes. Findings indicate that residents from both types of nursing homes experienced challenges to communicate their care needs and preferences in daily care activities. Moreover, residents or their representatives from both types of nursing homes demonstrated motivation and competence to master residents’ care based on their individual preferences, but also perceived that their motivation was not always supported by staff or the nursing home environment. Residents’ competence in mastering their care activities in ethno-specific nursing homes was based on the condition that they were given opportunities to use a language of choice in communication and staff and the nursing home demonstrated culturally competent care for them. In addition, ethno-specific nursing homes showed more recourse to support residents to maintain meaningful relationships with peers and others.

**Conclusions:**

Culturally competent care created by staff, nursing homes and the aged care system is a basic condition for residents from ethnic minority groups to fulfil self-determination. In addition, person-centred care approach enables residents to optimise self-determination.

**Supplementary Information:**

The online version contains supplementary material available at 10.1186/s12877-023-03800-w.

## Introduction

Supporting nursing home residents to fulfil self-determination has been described as enabling them to 1) exercise their autonomy to choose their care preferences; 2) demonstrate their competence to control their care; and 3) maintain meaningful connections with others [1]. Fulfilling self-determination is a basic psychological need for human beings [2]. Studies revealed that supporting residents fulfilling self-determination is positively associated with their health, wellbeing and quality of life [3, 4]. Residents from culturally and linguistically diverse (CALD) backgrounds in developed countries experience significant challenges in fulfilling self-determination compared to non-CALD residents in a mainstream nursing home due to known cross-cultural communication challenges with staff [5]. Moreover, residents’ preferences (i.e. diet) are strongly related to their lived experiences in their culture [6]. Therefore, care services designed for residents mainly from the mainstream culture may not meet CALD residents’ preferences. To overcome these challenges, ethno-specific nursing homes were established to provide culturally tailored services and employ bilingual and bicultural staff to match residents’ culture and language [7, 8]. Establishing ethno-specific nursing homes was driven by the problems identified in the social care system and was embraced by the ethnic minority groups [8, 9].

### Cultural and linguistic diversity in ethno-specific and mainstream nursing homes

Australia possesses ethno-specific nursing homes for some migrant groups from non-English speaking countries [8]. However, such an ideal care environment has been threatened in recent years due to difficulties in recruiting staff to match residents’ culture and linguistic background in the context of increased cultural and linguistic diversity in the aged care workforce where up to 50% of personal care workers are from various ethnic groups, mainly Southern Asian and African regions [8, 10]. Based on recent information, ethno-specific nursing homes in Australia now employ up to 48% of their staff from multiple ethnic groups [11] and provide services for up to 30% of their residents who are from other ethnic groups [8]. Meanwhile, mainstream nursing homes show increased cultural diversity evidenced by up to 48% of staff and 12% of residents from diverse cultural backgrounds [11]. The mismatch of culture and language between residents and staff in ethno-specific nursing homes were also reported in Canada and the United States (US) [12, 13]. However, it is not clear how residents cope with challenges posed by cross-cultural care in different types of nursing homes when fulfilling self-determination.

A few studies compared care outcomes for residents from ethno-specific and mainstream nursing homes and results were inconclusive [8, 14]. For example, one cross-sectional study reported that CALD residents living in ethno-specific nursing homes had more care needs met and less changed behaviours, which implied better care, compared to CALD residents living in mainstream nursing homes [14]. However, a more recent study reported that CALD residents living in ethno-specific nursing homes showed more unmet care needs and changed behaviours compared with non-CALD residents from the mainstream background living in the mainstream nursing homes using a national cross-sectional survey dataset [8]. These studies were unable to clarify staff and organisational conditions that contributed to the care disparities between resident groups due to the use of quantitative study designs.

### Factors affecting residents to self-determine own care

A large proportion of residents living in nursing homes have dementia [15]. Studies found that residents with dementia were still capable of engaging in some own care activities and their preferences remained stable over time [16]. Recent literature reviews on CALD residents in nursing home in developed countries found that their proficiency in mastering the dominant language of a particular country was a prerequisite for them to fulfil self-determination in everyday care [5, 15]. Studies showed that staff who did not share the same language and culture with residents in ethno-specific nursing homes could demonstrate culturally congruent care and prevent changed behaviours in CALD residents when they adapted their practices to residents’ culture, for example learning residents’ native language and using it when communicating with them, and respecting residents’ cultural values [12, 13].

In mainstream nursing homes, residents also faced challenges to fulfil their self-determination [17, 18]. The organisational factors such as no choice, predetermined choices and restricted choices for residents existed in food services, activities of daily living (ADLs) support, leisure and social activities existed in mainstream nursing homes [18]. Moreover, lack of opportunities for residents to freely communicate preferences and participate in care planning contributed to the unmet preferences [19]. As a result, residents’ sense of autonomy was undermined and satisfaction with care services was low [18]. Studies also identified staff factors affecting residents fulfilling their self-determination [20, 21]. For example, residents, who were cognitively intact, but had physical disabilities, showed frustrations due to delayed staff responses to requests for ADL assistance [20]. Staff’s unfamiliarity with residents’ care needs and preferences, views about job priorities and attitudes toward residents’ requests were attributed to unsupportive practices for residents in self-determining their care [20, 21]. In addition, residents also experienced cross-cultural communication challenges with CALD staff which affected them meeting care needs and preferences [22].

A study on ‘a preference-based model of care’ by Van Haitsma et al. [6] identified that residents’ preferences to connect with others was strongly influenced by their cultural values [6]. Therefore, residents from CALD and non-CALD groups may have different preferences for activities and events that help them interact with others. Moreover, interaction with peers was driven by residents’ motivation to identify those with similar interests [23]. Due to this, residents not sharing a similar culture and language may experience loneliness and maladaptation to the nursing home [23].

### Using person-centred care approach to optimise residents’ self-determination

It is well-recognised that diversity exists within the same cultural group [24]. Therefore, the person-centred care approach is a condition for individual residents to optimise self-determination [24]. Person-centred care is defined by the American Geriatrics Society Expert Panel in these aspects as described in the followings [24]. First, staff are required to demonstrate capabilities to elicit residents’ values and preferences and to provide care based on these values and preferences. Second, nursing homes are required to demonstrate policies, standards, procedures, resources, staff development activities and quality improvement activities that support person-centred care. In addition, the aged care system is required to demonstrate policies, resources, standards and regulations that support staff and nursing homes to provide person-centred care.

## Methods

### Aims

The aim of the study was to compare factors affecting residents fulfilling self-determination in ethno-specific and mainstream nursing homes.

### Study design

A qualitative descriptive approach was applied to address the study aim [25]. This design not only enabled the researchers to closely describe participants’ views on the areas of inquiry based on their lived experiences, but also to inform changes in care services through the lens of the service users’ [25, 26]. We followed the COREQ (see Supplementary file 1) in the report to foster the transparency of the study [27]. All methods were performed in accordance with the relevant guidelines and regulations.

### Ethical consideration

Ethics approval was obtained by Flinders University Social and Behavioural Research Ethics Committee (project number: 6841). All study participants provided written informed consent prior to their involvement. To ensure anonymity, all participants were assigned a participant number.

### Setting and participants

Participants were from a Greek ethno-specific nursing home, an Italian ethno-specific nursing home and a mainstream nursing home in an Australian state. The rationale for selecting these three specific participating nursing homes was based on our systematic review prior to study that residents from ethno-specific and mainstream nursing homes experienced different forms of challenges in fulfilling self-determination [5]. Moreover, these three nursing homes are non-for-profit nursing homes, receive Australian Government funding and are regulated under the same aged care standards [28]; therefore, enabled the project team to compare staff and organisational factors affecting residents to fulfil self-determination. In addition, residents from Italian and Greek backgrounds represent the first and third largest groups who were born in non-English-speaking countries in Australia respectively [8] which helped the project team to analyse the issues of concern associated with CALD residents.

Over 70% residents were Greek or Italian in the Greek ethno-specific and Italian ethno-specific nursing homes respectively. In these nursing homes, over 70% of direct care staff did not match the residents’ cultural backgrounds but were from the mainstream Australian culture or south Asian and African regions. In the mainstream nursing home, most residents were from the mainstream Australian culture and a small proportion (up to 15%) of residents were from other cultural backgrounds, including Filipino, Chinese, Spanish, Greek and Italian who were the first-generation immigrants based on the resident profile provided by the nursing home. In this facility, over 40% of care staff were from south Asian and African regions and the rest were from the Australian culture. Information about residents and staff cultural and linguistic diversity was provided by the three nursing homes in the grant application for a partnership grant scheme to support the study.

Purposive sampling method was used to recruit participants who were able to share their lived experiences in the study area. The inclusion criteria were: (1) permanent residents who had lived in the residential care facility for at least 3 months; and (2) family members were invited if residents had cognitive impairment. Family members were also welcome to join interviews with residents without cognitive impairment if the resident requested. An interpreter was arranged for residents who were unable to speak English.

### Data collection

We provided residents and their families with face-to-face information sessions to introduce the study, invited them to the study and answer any questions they may have. We also provided written information about the study to them which explained the study, voluntary nature of participation and the way to maintain confidentiality of information for participants. Residents and their family members who were willing to participate in the study were encouraged to express their interest by sending a phone message, email or posting an expression of interest slip via a pre-paid envelope to a researcher (CG).

Interviews began in March 2020 and ended in April 2021 due to COVID-19 restrictions. Mostly individual interviews were conducted with residents and family members. However, a focus group was offered as an alternate option to participants. A semi-structured interview guide was developed based on our literature review in the project to guide the interviews and focus group (see Table 1). Individual interviews were conducted by the second author (CG) in residents’ rooms, a private area of the nursing home or online when COVID-19 lockdown measures prevented visiting in person. The focus group was conducted in the facility’s gym by the second author and a bilingual assistant who acted as an interpreter and moderator. Interviews were digitally recorded. Demographic information was collected beforehand. Data collection was ceased when no new information regarding the aim of study was found from the interviews.

**Table 1:** Semi-structured interview questions

1. Can you please tell me the country where you were born?
2. Did you have regular contact with ethnical or/and religious community organisations before moving into the care home? a. What has changed about your contacts with ethnical or/and religious community organisations since you moved into the care home? b. What changes could be made so that you could maintain contact with ethnical or/and religious community organisations?
3. Please tell me the main food you had at home a. What has changed about what you eat since you came to the home? b. What changes could be made so that you could get the food you like to eat?
4. Please tell me your leisure activities and hobbies a. What has changed about what your leisure activities and hobbies since you came to the home? b. What changes could be made so that you could maintain activities and hobbies you still enjoy?
5. Do you have any preferences, likes and dislikes in everyday care that are related to your ethnical background, your culture and religious beliefs? If yes, a. Please tell me these preferences, likes and dislikes b. How do you let staff know and meet these preferences, likes and dislikes? c. What special considerations or/and actions do staff take to meet these preferences, likes and dislikes? Please give some examples
6. Have you experienced situations that your preferences, likes and dislikes in everyday care were not met? If yes, what do you do to let staff meet these preferences, likes and dislikes?
7. Have you experienced situations that your care needs are not met because you cannot speak English or cannot speak English well? If yes, what do you do to let staff understand and meet your care needs?
8. What are your wishes to control everyday care activities, for example to make decision and control what activities to be included; how to deliver those to you; and what time to deliver those to you? a. Have you discussed these wishes with staff? b. If yes, what do staff do to consider and meet your wishes?
9. When you are cared for by staff from other ethnic/cultural group, is it easy or difficult to let them understand and meet your care needs and preferences? a. If it is easy, please give some examples b. If it is difficult, what do you do to let the staff understand and meet your care needs and preferences?
10. When you are cared for by care staff from other ethnic/cultural group, have you experienced difficulties in understanding what the staff said to you? a. If yes, what helped you to understand them? b. What suggestions do you have for the care home to improve this situation?
11. If your relative has dementia, what do you do to help staff to understand and meet the care needs and preferences of your relative?

### Data analysis

Interview data was transcribed verbatim for analysis. Data were entered into NVIVO Version 12 for the management of data analysis. The first and second authors (LX and CG) undertook the data analysis independently using the thematic analysis described by Nowell et al. [29]. First, the researchers read and re-read each transcript to be familiar with interview data. Second, they undertook open coding to identify meaningful words related to the study aim from each transcript. Cross-checking of coding by the research team was undertaken to avoid bias. Third, they grouped codes based on similarities and describe the group codes. Fourth, they related the group codes with the study aim to conceptualise potential sub-themes and themes. Finally, they reviewed these sub-themes and themes by relating quotes from interviews/ focus group to ensure the consistence between the results and the data. All team members including the representatives of industry partners were given opportunities to review and comment on the results. Differences were resolved through discussions in regular steering committee meetings of the study.

### Study rigor

The study is in line with credibility, confirmability, dependability and transferability of qualitative research [30]. Credibility was achieved through team members’ long-term engagement with the three participating organisations even before the present study (LX and CG) which helped us build trust with participants. All authors have a track record in qualitative studies. At the end of each interview, the researcher (CG) drew main points on each interview question and asked participants if these represent their view and if they would like to add any points. The researcher also took field notes during the interview to document non-verbal communication that helped with confirmability in data analysis. We also undertook member checking throughout the data analysis and in the written report to ensure the consistence between data and results and to avoid biases. We used excerpts from interviews to present results. These processes helped us enhance the confirmability. We demonstrated dependability or an audit trail by showing step-by-step data analysis. We also provided detailed description about the study context and rich information on findings which enable the transfer of findings to similar socio-cultural context (transferability).

## Results

In total, 29 participants, including 24 residents and 5 family members whose relatives had dementia, participated. Interviews lasted an average of 35 min (20–60 min) while the focus group was 90 min. The short interviews with some residents were due to their choice to discuss questions they were interested in. Among the 29 participants, 16 were from ethno-specific nursing homes (8 Italian residents, 4 Greek residents, one Australian-born resident and three family members) and 13 were from the mainstream nursing home (10 Australian-born residents, one from CALD background who spoke English in the interview and 2 family members) (see Table 2). Only four residents from Greek background in the interview and Italian residents in the focus group received interpreter assistance. The mean aged of residents was 86.3 (range 80–98) years old and the mean length of stay in nursing homes was 18.8 (ranging 3–60) months. The mean length of stay in Australia for overseas-born residents was 57.4 (ranging 1.5–68) years. The detailed demographic information about participants is presented in Table 2.

**Table 2:** Socio-demographic Information of Participants

Code	Participant	Gender	Age	Ethnicity	Nursing home types	Months in the nursing home	Years in Australia	Data collection types
1	Residents	Female	91	Australian	Mainstream	60	NA	Interview
2	Residents	Female	80	English	Mainstream	36	56	Interview
3	Residents	Female	80	Australian	Italian ethno-specific	12	60	Interview
4	Residents	Male	75	Australian	Mainstream	18	NA	Interview
5	Residents	Male	85	German	Mainstream	8	56	Interview
6	Residents	Female	88	Australian	Mainstream	18	NA	Interview
7	Residents	Female	93	English	Mainstream	5	1.5	Interview
8	Residents	Female	98	English	Mainstream	45	NA	Interview
9	Residents	Female	82	Australian	Mainstream	12	NA	Interview
10	Residents	Male	89	English	Mainstream	5	67	Interview
11	Residents	Male	88	English	Mainstream	9	68	Interview
12	Residents	Female	86	Greek	Greek ethno-specific	12	56	Interview
13	Residents	Female	82	Australian	Mainstream	10	NA	Interview
14	Residents	Female	85	Greek	Greek ethno-specific	14	56	Interview
15	Residents	Male	80	Greek	Greek ethno-specific	15	58	Interview
16	Residents	Male	85	Greek	Greek ethno-specific	10	56	Interview
17	Residents	Female	82	Italian	Italian ethno-specific	60	53	Focus group
18	Residents	Female	93	Italian	Italian ethno-specific	6	68	Focus group
19	Residents	Female	94	Italian	Italian ethno-specific	36	68	Focus group
20	Residents	Female	88	Italian	Italian ethno-specific	24	57	Focus group
21	Residents	Male	86	Italian	Italian ethno-specific	12	58	Focus group
22	Residents	Female	85	Italian	Italian ethno-specific	12	61	Focus group
23	Residents	Female	87	Italian	Italian ethno-specific	8	65	Focus group
24	Residents	Female	89	Italian	Italian ethno-specific	3	68	Focus group
25	Daughter	Female	62	Greek	Greek ethno-specific	NA	NA	Interview
26	Daughter	Female	58	Australia	Italian ethno-specific	NA	NA	Interview
27	Daughter	Female	60	Australia	Italian ethno-specific	NA	NA	Interview
28	Daughter	Female	61	Australia	Mainstream	NA	NA	Interview
29	Husband	Male	82	English	Mainstream	NA	56	Interview

Code number 25–29 represent family members participated in the study

Three main themes were identified and described as communicating needs and preferences; mastering own care; and maintaining meaningful relationships. Each theme includes sub-themes that detailed similarities and differences of factors affecting residents fulfilling self-determination in the two types of nursing homes. We use ‘RFG’ to represent the resident focus group, and ‘ETHNO’ and ‘non-ETHNO’ to represent whether residents were from an ethno-specific nursing home or the mainstream nursing home.

### Theme 1: Communicating needs and preferences

#### Sub-themes identified in the ethno-specific nursing home

All resident participants in ethno-specific nursing home experienced cross-cultural communication challenges which required staff to demonstrate culturally competent communication. Residents also expected person-centred care approach as detailed in the follows.

##### The need for assistance in cross-cultural communication

In ethno-specific nursing homes, all residents described the need to use a language of choice to express their preferences: ‘I am Italian. My language is Italian. There is not much I can do about that. But if they[staff] don’t understand, they will find someone who speaks Italian to interpret and/or they will ring up my daughter’ (RFG_ETHNO). Such a situation underscored staff and the nursing home to create an ideal social condition for residents to use the language of choice to freely express their care needs and preferences.

The lack of opportunities to learn English in their early life of migration in Australia were attributed to the need to have translation services in nursing homes:

Unfortunately, none of us went to school to learn English. That wasn’t our thing. None of us went to learn English. Some of us speak a little bit of English because we may have worked in the workforce… But there is not many of us that can say that we speak English especially when it is necessary. When it is necessary, we find somebody here to translate (RFG_ ETHNO).

As described above, having culturally competent bilingual staff to provide interpreter services was a basic condition for CALD residents to meet their care needs and preferences in ethno-specific nursing homes where a large proportion of staff did not share the same language and culture with them.

##### Valuing designated bilingual staff

Residents highly valued the interpreter services provided by designated bilingual staff appointed by the nursing home:

When there is something I don’t understand, I go to an Italian speaking staff member and ask them. There are a couple of carers that work in the evening that do understand Italian. So I do try and find someone who speaks Italian (RFG_ETHNO).

The availability of a designated staff was also observed by a family member: ‘But there is Greek speaking staff as well. So, there's always somebody in the main area that spends a bit of time with them’ (#25 Relative_ETHNO).

##### The need for timely assistance in cross-cultural communication

Residents mentioned that they could not access bilingual staff at the time they needed: ‘I wish that there would be more Italian speaking staff here because I do not speak English and I would be more happier if there were more Italian speaking staff’ (RFG_ ETHNO). Residents also encountered staff who showed little willingness to help them due to cross-cultural communication challenges: ‘Some carers will say that they don’t understand me, and they will go’ (RFG_ETHNO). Therefore, they suggested: ‘They should go and learn Italian’ (RFG_ ETHNO). Such a situation underscored the nursing home’s responsibility to provide education for staff to develop cultural competence.

Residents also mentioned that their family assisted them to communicate their needs and preferences: ‘If they[staff] don’t understand they will ring up my daughter’ (RFG_ETHNO). This situation indicated that bilingual staff although available were not always accessible. The need to have timely access to bilingual staff for help indicated that more person-centred care approach to interpreter services for each resident was much needed.

#### Sub-themes identified in the mainstream nursing home

Resident participants in this type of nursing home mainly experienced challenges associated with person-centred care. Occasionally, they also experienced communication challenge that was associated with culturally competent care as detailed in the follows.

##### The impact of functional impairment on communication

In the mainstream nursing home, non-CALD residents mainly mentioned that communication barriers was due to staff’s lack of awareness of the impact that their cognitive decline had on communication: ‘I don’t understand when people talk fast as it takes me a while to comprehend’ (#6 Resident_non-ETHNO). The finding indicated that residents’ preferences in communication were not assessed or communicated by staff, and staff did not act in accordance with residents’ communication preferences.

Residents’ family members also raised a concern about staff’s capability of assessing residents with dementia:

I think that people with dementia have very specific issues that are extremely difficult for both carers and nurses to understand if they're not with that person for a long period of time and when they have a major health problem sometimes’ (#28 Relative_non- ETHNO).

This concern indicated that person-centred communication approach was a condition for staff to assess and meet care needs and preferences for residents living with dementia.

##### Cross-cultural communication challenge with CALD staff

Non-CALD residents experienced cross-cultural communication challenges with new CALD staff: ‘The only problem we have with the staff is that agency staff…Occasionally they can't speak English. You keep repeating it over and over again until you seem to get through either by hand signals or by word of mouth’ (#2 Resident_non-ETHNO). The cross-cultural communication challenges with CALD staff were also observed by a resident’s family member:

Um, I’m there a lot and observe a lot of staff with other cultural backgrounds mixture of Philippine, African and India. Main problem is the accent…and mum would say, Oh yeah, sorry, I didn't understand that and they will repeat it, but it would be exactly the same (#28 Relative_non- ETHNO).

Such cross-cultural communication challenges were mainly attributed to staff’s low English proficiency which underscored the selection criteria for CALD staff. These situations also indicated the need to have local staff to mentor CALD staff in cross-cultural communication in the care team to create a culturally competent care condition for residents.

### Theme 2: Mastering own care

Resident participants or their family members in both ethno-specific and mainstream nursing homes demonstrated their competence to master residents’ care activities. They expected nursing homes to meet their individual preferences via a person-centred care approach. However, this was based on the condition that CALD residents were given opportunities to use a language of choice in interactions with peers and staff which indicated culturally competent care as a basic condition as detailed in the follows.

#### Sub-themes identified in the ethno-specific nursing home

##### Wanting to feel at home

Residents perceived ‘Nothing is better than your own home’ (RFG_ETHNO). Food is a main concern when moving into nursing homes, as one stated: ‘The quality of the food is not good, no tasty…It is very difficult for me here because I have been all these years with my husband cook everything, nice and healthy’ (#12 resident_ETHNO). Therefore, residents were motivated to work with staff to make the facility more homelike through their active participation in quality improvement forums using a language of choice: ‘We asked menu choices today for tomorrow and the next day. Sometimes we don’t get what asked for’ (RFG_ETHNO).

Residents also perceived that sometimes their individual dietary preferences were not addressed: ‘First when I came, I ask kitchen if they could do XX (Particular type of Italian soup) every now and then. They said they could not do it cos not many people like it’ (RFG_ETHNO). This resident experience indicated that ethno-specific nursing homes still face challenges to meet residents’ individualised dietary preferences. Therefore, creating resources or new services to enable person-centred care was imperative. Unmet individual dietary preferences were also mentioned by another resident: ‘I have asked many times about having green leafy vegetables…That is the big thing because that is what we are used to’ (RFG_ ETHNO). This case indicated that that staff were aware of the resident’s individual dietary preference, but did not act to meet the preferences, an indicator that person-centred care approach need to be incorporated to daily care activities.

Residents expected to maintain their lifetime hobbies or lifestyles when living in nursing homes but perceived that support for them to achieve this goal was limited: ‘Here nobody’s asked me to go in the garden. …there’s a couple of people that look after the garden and I don’t think they would like outsiders to go into their garden’ (#14 resident_ ETHNO). The lack of individualised support for residents to maintain their lives similar to those at their homes was echoed by a resident’s family member: ‘Mom's just always been fastidious with her cleaning and she likes to feel valued. And if you gave her a job, she'd gladly carry it out. So I think she probably misses that side of things’ (#26 Relative_non- ETHNO).

##### The impact of high-dependence on mastering own care

Residents with dementia may lose their ability to master their care, as a family member described:

Mum has some memory issues. …I came to visit and she had a really bad headache and she'd said that she skipped lunch but she hadn't actually asked anyone. So, I do think that they [staff] should probably be a bit more vigilant in really making sure that she is okay (#26 Relative_ETHNO).

In this case, the resident with dementia largely relied on family members to master their care needs and services. This situation indicated the lack of proactive actions from staff to monitor and assess residents with dementia using person-centred care approach.

#### Sub-themes identified in the mainstream nursing home

##### Wanting to feel at home

Residents in the mainstream nursing home also would like master their lives similar to those when they lived at own home: ‘I like to have a couple of glasses of wine, white wine. And the doctors got me on one at lunch time and two at night’ (#9 Resident_non-ETHNO). However, it seemed that they had to initiate a request and gain permission from others to meet the desire for preferred lifestyle.

Residents communicated with staff about their individual dietary preferences but did not receive any response: ‘I like things that are either roasted or grilled…We have a roast once a week. We don't have any grills…You mention things and they don’t get done so you might as well keep quiet’ (#8 Resident_non-ETHNO). Another resident echoed similar frustration: ‘Well, I've learned to eat rice and. I used to use it as a pudding, but not any other way. So, I've learned to eat it. Otherwise, you have to be hungry’ (#9 Resident_non-ETHNO). Those situations contributed to need frustration for residents which have a negative consequence on their mental health. These cases also indicated the lack of assessment of residents’ values and preferences underpinned by person-centred care.

Residents was also capable of taking proactive actions to meet their preferred exercises when such exercises were not available in the nursing home: ‘I don't worry greatly about their exercise classes they have here because I've got my own equipment. And I do daily exercises myself. I've got a pedal machine. I've got weights to strap on my ankles and arms’ (#4 Resident_non- ETHNO).

Several residents divulged that they managed ‘secret activities’, which indicated the lack of sense of home in the nursing home as a resident said:

‘I am a Buddhist hermit and none of the staff knows that…I take steps to hide my practices, for example meditating in a chair with a novel so that anyone coming in would assume that I am reading. I also meditate in bed at night before going to sleep’ (#10 Resident_non-ETHNO).

Another resident shared similar experience: ‘I’m in a chair so I read, I sew, I knit and occasionally I write poetry. That’s a secret’ (#7 Resident_non-ETHNO). When the researcher asked: ‘What, you don't want to share it with anybody?’ the resident responded: ‘No, no, no.’ (#7 Resident_non-ETHNO). These cases indicated that residents had to spend energy to protect their privacy in the nursing home, an indicator of low-level of sense of home.

##### The impact of high-dependence on mastering own care

Residents with physical disability felt frustrated when care was not delivered in a timely manner: ‘I have got to have them put me on the toilet. And I can ring the bell. And I have waited half an hour…I'm just gonna sit and wait and accept that’ (#4 Resident_non-ETHNO). This case may indicate inadequate staffing level or a low awareness in staff about individual residents’ care needs and preferences. The tolerance of such an unsatisfactory care also indicated resident perceived vulnerability and a fear of reprisal.

Residents with dementia relied on family members to master their care through daily frequent visits to the nursing home: ‘Well, she wouldn't know what to choose anyway. Because of her dementia. … She answers certain questions sometimes, but you can't have a conversation with her’ (#29 Relative_ non- ETHNO). When the researcher asked: ‘So how do you let the staff know about her preferences and the things that she might like or dislike?’, the answer was: ‘Well, it's not really discussed’ (#29 Relative_ non- ETHNO). This case indicated the need to incorporate person-centred care approach to the daily care activities for residents with dementia.

### Theme 3: Maintaining meaningful relationships

Resident participants in both ethno-specific and mainstream nursing homes desired to maintain meaningful relationships based on their individual preferences which required person-centred care approach. CALD residents were given opportunities to use a language of choice in connecting with others in cultural and religious activities which indicated culturally competent care as a basic condition as detailed in the follows.

#### Sub-themes identified in the ethno-specific nursing home

##### Desire to connect with families

Connecting with family members was highly valued by residents: ‘We had lots of visitors…I have three daughters and the grandchildren. The home full all the time… There was always somebody popping in. Now not much because of the corona virus’ (#12 Resident_ETHNO). This case indicated residents’ unmet preferences in connection with family members during the COVID-19 restrictions. This situation may also indicate that nursing homes had limited resources to support residents to interact with their families which was confirmed by a resident’s family member:

Mum can’t use a mobile because she has never used one before and she's just getting to the stage where she is just getting a little bit forgetful. So, we only rely on phone calls when we call up during the week. But if that was available through WhatsApp or through Facebook or Zoom, that would be nice (#25 Relative_ETHNO).

Even COVID-19 restrictions were lifted, residents perceived that support from the nursing home for family visitors was limited: ‘Now we can have visitors come to our unit but not into community space. We would like visitors to be able to come into the community room. X [manager] is aware of this… hope to allow very soon’ (RFG_ETHNO). These situations indicated that residents expected more individualised support for them to interact with their family members using person-centred approach.

##### Desire to interaction with peers

Residents would like to have prolonged interactions with peers: ‘After dinner everybody goes into their own room…the time is too long to wait to go to sleep…it would be good if we could be together a little bit longer. It is a long time to be alone by yourself’ (RFG_ETHNO). Such a request may indicate loneliness. The lack of interactions with peers was also described a resident’s family member:

But now she does a lot of knitting and she crochets, but she sits in her room and does that a lot because there's not many other ladies that are of the same ability. If there was a small group of residents that were of like ability, mentally and physically I guess, where she could socialise and like have a knitting club’ (#25 Relative_ETHNO).

The unmet desire for individulised interactions with peers may be an indicator of resource limit in the nursing homes.

##### Onsite religious activities bringing people together

Residents were still very connected to religious and community activities and events they had been involved with throughout their life. Onsite church was available in the two ethno-specific nursing homes for residents to socialise with their peers who shared the same religion, as a resident said: ‘It is good we have masses in Italian more than once a week’ (RFG_ETHNO). They also observed that ‘Church attendance has increased’ (RFG_ETHNO). Residents also described that, ‘The Sisters of Saint Anthony provide pastoral and spiritual care’ (RFG_ETHNO). Those activities indicate the ethno-specific nursing homes’ connections with community organisations by which they were able to provide resources needed to meet residents’ expectations for maintaining their religious activities they had through their live course.

#### Sub-themes identified in the mainstream nursing home

##### Desire to connect with families

For residents with dementia, family members played a key role in maintaining their connection with meaningful relationships: ‘But the problem is now she's with her dementia…So she's isolated in her room and this is very much dependent on her family visit each day (#28 Relative_non-ETHNO). This case indicated, in the absence of family members, residents with dementia experienced social isolation. This case also indicated that the resident’s preferences in connecting with others might not be assessed or not be addressed due to staffing issues.

Residents also perceived that COVID-19 had a profound impact on them to interact with their family:

It is very difficult with this virus hanging over us…we are confined. But hopefully when we can get out ... my daughter will come and take me out for the afternoon for a ride round. Well, that will be fine for me to visit her at home (#7 Resident_non-ETHNO).

A resident’ family echoed similar perception: ‘Now, I can't because of the change with the COVID situation’ (#28 Relative_non-ETHNO). Addressing the preferred interactions with family members require person-centred approach which had an implication for resources to support this approach.

##### Desire to interact with peers

Group activities were highly valued by residents. Some residents join in activities just for the purpose of socialising with peers and overcoming loneliness: ‘Well, everything's all right as long as you are not sitting in your room twiddling your thumbs…I'm not a big bingo fan, but I think we're offered and it's not costing anything. And it gets to know people’ (#6 Resident_non-ETHNO). This case indicated that individual values and preferences were not assessed, and leisure activities provided by the nursing home did not meet residents’ individual preferences. However, residents also demonstrated proactive actions to meet their individual preferences in interactions with others:

Before moving [into the nursing home], I had a very active life and was always busy. I volunteered at the nursing home for many years. I walk around the corridors three times a day for exercise and to talk to other people. I don’t like to spend a lot of time alone in my room during the day (#6 Resident-non-ETHNO).

##### Difficulties in attending church services

Onsite churches matching residents’ religious beliefs were not always available in the mainstream nursing home: ‘I’m a Baptist. No, I’ve had no contact with Baptist Church here’ (#7 Resident_non-ETHNO). A resident’s family member echoed similar experience: ‘Yes, she regularly attended with the Anglican Church before moving into here… Well, my mum no longer has any regular contact with the church’ (#28 Relative_ non-ETHNO). This finding in the mainstream nursing home was contrast with the rich religious activities identified in ethno-specific nursing homes. The finding indicated that the mainstream nursing home may have limited resources or networks with community organisations to bring religious services to the nursing home to meet residents’ individual preferences in engaging religious activities.

## Discussion

Our findings add new knowledge to the literature regarding similarities and differences of residents and their family members perceived factors affecting residents fulfilling self-determination in ethno-specific and mainstream nursing homes as discussed in the following sections.

### Culturally competent care as a basic condition for residents to fulfil self-determination

Our findings support two previous literature reviews which found creating cultural and linguistic congruent care is a social condition for residents to communicate their needs and preferences and fulfil autonomy [5, 15]. However, our study revealed that arranging bilingual staff to be available and accessible for residents during all shifts was not always possible in ethno-specific nursing homes. Therefore, these ethno-specific nursing homes differed greatly from the ethno-specific nursing homes reported in Sweden where bilingual staff were employed to match residents’ culture and language use to provide culturally and linguistically congruent care for Finnish residents [7, 31]. Our findings also support the scoping review by Martin et al. [15] that the absence of culturally and linguistically congruent care conditions contributed to unmet care needs and dissatisfaction with care services. To avoid such negative consequences, the resident-bilingual staff ratio needs be carefully examined and incorporated into policies and regulations in the aged care system. Ideally, a large proportion of bilingual staff should be employed to provide direct care for residents [32]. As ethno-specific nursing homes have increased numbers of staff from a culture other than the residents’, staff education on culturally competent care and teamwork are imperative.

In the mainstream nursing home, our study revealed that staff’s lack of knowledge and skills in communicating with residents with cognitive decline affected these residents’ ability to discuss and negotiate their care needs and preferences. This finding supports previous studies that staff showed a low level of awareness of residents’ functional impairments and the negative impact on their communications [19, 33]. A large cross-sectional study confirmed that residents with sensory impairment had more unmet preferences in daily care [34]. Our finding that residents in mainstream nursing homes experienced cross-cultural communication difficulties with staff from a non-English speaking background supports previous studies [22]. The finding further underscores staff education preparation in cross-cultural communication with residents to support them to fulfil autonomy.

### Supporting residents’ competence in mastering their own care

In our study, residents and family members in ethno-specific nursing homes showed active participation in quality improvement forums to improve residents’ diet choices. This finding supports the study by Paque et al. [35] who described that by nature residents were capable of voicing their preferences, a form of practical autonomy. However, our finding differed from a previous study on ‘Family Councils’ in two non-ethno-specific Canadian nursing homes which were established for family members to voice their opinions on quality improvements [36]. In that study, Chinese family members showed reluctance to participate due to a fear of ‘voicing their true opinions’ in their culture and language barriers [36]. In our study, the concentration of Italian and Greek residents in the two ethno-specific nursing homes and the use of their first language in quality improvement forums may encourage their collective action. However, we also found that residents with dementia relied on family members to monitor their conditions and advocate for them. Our finding supports a systematic review that described family involvement in residents’ care through frequent visits and associated such visits with improved health, wellbeing and quality of life for residents [37]. In another qualitative study, researchers found when staff showed intuitive knowledge they knew changes in residents and intervened even without residents’ initiation [21].

Our findings on residents’ acceptance of poor care services, for example sitting on the toilet to wait for staff to help or giving up their efforts when requests for preferred diet were rejected, are in line with previous studies [34, 38]. There may be two reasons for residents accepting poor services. First, residents had knowledge about the busy working environment and showed empathies for overloaded staff [38]. According to the study by Ferrand et al. [39], suppressing motivation for self-determination due to external forces contributed to frustration and depressive symptoms in residents. Second, residents, especially those with high-level dependence on staff, were aware of power relations with staff; therefore, they had a fear of repercussion if they complained [38]. Providing person-centred care for this group of residents requires adequate staff allocation, education for staff and empowering residents and their family members in quality improvements.

### Maintaining meaningful social interactions

Our finding that family played a main role in maintaining residents’ social engagement, supports previous studies which described social isolation experienced by residents with very limited nonfamily network in nursing homes, especially those with dementia [40, 41]. It is well-recognised that dementia affects residents’ cognitive function to comprehend information about activities or communicate with staff and peers about their needs [42]. Therefore, they are at a high risk of having unmet needs regarding relatedness to others which would result in poor quality of life [43]. Person-centred care approaches for this group of residents are particularly essential, but requires adequate staffing level, staff education and resources.

Our finding that family visitors were unable to use meeting rooms to interact with their loved ones after COVID-19 restrictions eased indicated that nursing homes had individual policies in COVID-19 prevention. The finding is in line with a qualitative study that compared Dutch and UK nursing home visiting during the COVID-19 pandemic in which family members faced some restrictions accessing rooms and spaces and visiting experiences were less enjoyable [44]. The findings suggest that nursing homes are required to create spaces and resources to support person-centred family interactions.

Our findings revealed that ethno-specific nursing homes possessed richer resources to support residents to maintain meaningful interactions with peers and others through religious activities and spiritual care compared to the mainstream nursing home. This finding supports studies on Finnish ethno-specific nursing homes in Sweden where rich resources to support residents’ social engagement were developed through connections and collaborations with community organisations [7, 31]. This finding also supports the suggestion that arranging residents from the same ethnic group in a unit of a mainstream nursing home, may be logistically viable in terms of encouraging socialisation among residents while developing resources to support culturally and linguistically congruent care [5, 7].

We identified that residents in both types of nursing homes desired more opportunities to interact with peers through structured activities and incidentally. The finding supports previous studies that residents lost their pre-existing social relationships when moving into the nursing home and they were motivated to establish new relationships [38, 45]. Developing friendships with peers is an indicator of adapting to the care environment [23]. A study by Roberts [45] identified at least three types of relationships that had a positive psychosocial impact on residents: acquaintanceship (i.e. friendly greetings between residents), casual friendships (i.e. mutual sharing of interests and enjoyable things) and close friendships (i.e. friendships for a lifetime which usually existed outside of the nursing home). In our study, most relationships were acquaintanceship and casual friendships.

### Limitations

Our study has some limitations. First, we only recruited one resident from an ethnic minority group who lived in the mainstream nursing homes. Therefore, findings from the mainstream nursing home cannot represent residents from the ethnic minority group who live in this type of nursing home. Second, we also only recruited one resident from the mainstream culture who lived in an ethno-specific nursing home. Therefore, findings from the ethno-specific nursing homes cannot represent this resident group. Due to those reasons, we did not include quotes from these two residents to support our findings. Third, our study recruited a low number of family members. Findings may not represent family members’ views.

## Conclusions

Residents from an ethnic minority group cared for in ethno-specific nursing homes, experience more cross-cultural communication challenges when expressing their care needs and preferences and exercising their competence to control their care activities, compared to residents from the mainstream culture cared for in a mainstream nursing home. However, they engage in more religious activities and spiritual care which gives them more opportunities to connect with peer residents compared to those living in a mainstream nursing home. In both types of nursing homes, residents with dementia mainly rely on family members to assist them to fulfil self-determination in daily care activities. Both types of homes need to work in partnership with policymakers, community organisations, residents and their families to develop resources, staff capacity and capabilities that enable a home-like environment for residents to exercise their autonomy in everyday care activities.

## Supplementary Information

**Additional file 1: Supplementary file 1.** COREQ.

## Data Availability

The data (interview transcripts) used in the study cannot be deposited publicly due to confidentiality of information but are available from the corresponding author on reasonable request.

## Abbreviation

CALD: Culturally and linguistically diverse
